# Effects of Codon Usage on Gene Expression: Empirical Studies on Drosophila

**DOI:** 10.1007/s00239-015-9675-y

**Published:** 2015-04-03

**Authors:** Jeffrey R. Powell, Kirstin Dion

**Affiliations:** Department of Ecology and Evolutionary Biology, Yale University, 21 Sachem Street, New Haven, CT 06520-8105 USA

**Keywords:** Codon usage bias, *Drosophila*, Gene expression, Translation

## Abstract

For most amino acids, more than one codon can be used. Many hypotheses have been put forward to account for patterns of uneven use of synonymous codons (codon usage bias) that most often have been indirectly tested primarily by analyses of patterns. Direct experimental tests of effects of synonymous codon usage are available for unicellular organisms, however empirical data addressing this problem in multicellular eukaryotes are sparse. We have developed a flexible transfecting plasmid that allows us to empirically test the effects of different codons on transcription and translation and present data from *Drosophila*. We could detect no significant effects of codon usage on transcription. With regard to translation, optimal codons (most used) produce higher levels of protein expression compared to non-optimal codons if the effect of difference in thermodynamic stability of secondary structure of the 5′ mRNA ribosome-binding site is controlled for. These results are consistent with what has been found in bacteria and thus expand the generality of these principles to multicellular eukaryotes.

**Electronic supplementary material:**

The online version of this article (doi:10.1007/s00239-015-9675-y) contains supplementary material, which is available to authorized users.

## Introduction

The genetic code is redundant: for most amino acids, more than one codon can be used to code for the same amino acid in a protein sequence. All other factors equal (such as random mutation), it is expected that over time (at equilibrium) codons for the same amino acid would be used equally frequently. But that is not the observation: codon usage is usually biased with one or two codons for an amino acid being used much more frequently than others. Which codon(s) is favored varies from taxon to taxon, that is, there is no universal pattern of codon usage bias (CUB). Why this is the case has interested molecular evolutionists for decades. Many hypotheses have been put forward to account for these observations (reviewed in Plotkin and Kudla 2011). For example, mutation bias has been given support for causing CUB in warm-blooded vertebrates that have “isochore” structured genomes (Aota and Ikemura 1986; Bulmer 1988). However, in some organisms such as *Drosophila* that have no isochores, mutation bias is toward A/T while codon bias is toward codons ending in G/C (Powell and Moriyama 1997), i.e., mutation pressure is away from the most used codons. Thus other factors such as selection must be in play.

Most selection-based explanations of CUB have focused on the effects of codon usage on translation. It was hypothesized long ago that isoaccepting tRNAs (tRNAs that have the same amino acid but different anti-codons) may be in unequal abundance in the cytoplasm and this may affect efficiency of translation of different synonymous codons (Zuckerkandl and Pauling 1965; Richmond 1970). This was given support when it was documented that the most abundant tRNAs decoded most efficiently the most used codons in bacteria and yeast (Ikemura 1981, 1982) and that genes with higher protein expression had higher CUB (Grantham et al. 1981). In addition to speed of translation resulting in higher protein expression, Akashi (1994) provided evidence that accuracy of translation may also be involved; the most used codons result in lower levels of mis-incorporation of amino acids. The term “efficiency” of translation can be used to encompass both speed and accuracy.

In addition to affecting translation *per se*, codon usage may have effects on RNA structure that can affect other processes. Efficiency of splicing is one such process (Parmley and Hurst 2007). Another is the rate of mRNA ribosome binding. In bacteria, synonymous codons that change the thermodynamic stability of the secondary structure of the 5′ ribosome binding site of mRNA has been found to effect levels of protein expression; stronger secondary structure results in lower protein production (Kudla et al. 2009; Goodman et al. 2013).

Empirical tests of hypotheses accounting for CUB have come mostly from single-celled organisms, bacteria and yeast (Plotkin and Kudla 2011). Direct experimental test involving multi-cellular eukaryotes includes work by Carlini and Stephan (2003) and Hense et al. (2010) on the *Drosophila* alcohol dehydrogenase gene (*Adh*) and Lampson et al. (2013) on oncogene expression in human tissue culture cells.

We have developed a transfecting plasmid with cloning sites that allows us to insert a synthetic oligonucleotide of our choice and measure its effect on both transcription and translation. We tested how codon usage affects both transcription and translation in *Drosophila* cells including examining the role of stability of the secondary structure of the ribosome binding site. The plasmid also works in human cells and therefore potentially provides a general way to test hypotheses in a broader range of organisms.

## Materials and Methods

### Materials and Protocol

**Fig. 1:**
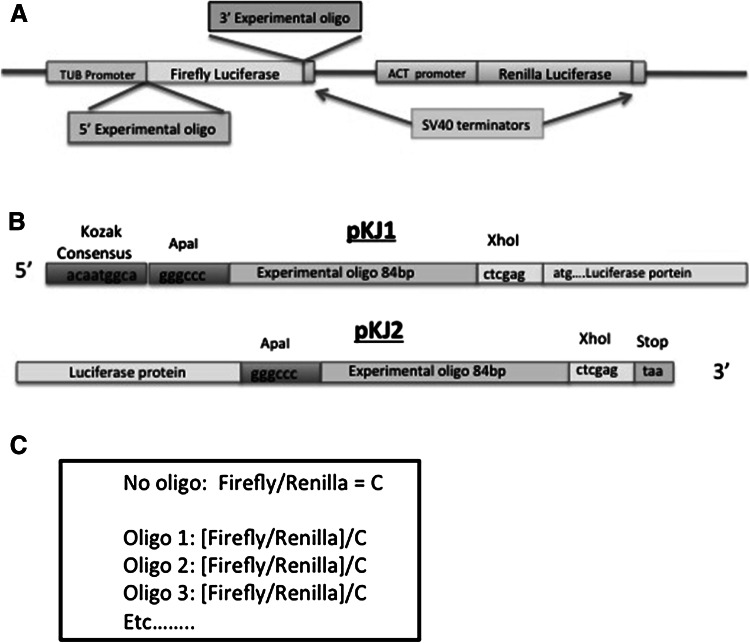
**a** Schematic of plasmid used in experiments. The plasmid is modified from pRL-null vector (Promega) as detailed in materials. Two cloning sites, one at each end of the protein-coding firefly luciferase gene, are shown. Distances and lengths of sequences are meant to convey general configuration and not actual distances or sizes; total length of plasmid is 10.5 Kb. **b** Details of the insertion sites. **c** Data are handled as indicated in the *box*, that is, the ratio of reporter expression is normalized to the control with no inserted oligo

The experimental plasmid (Fig. 1a) was derived from the commercially available pRL-null vector (Promega, Inc.). The luciferase and SV40 region were obtained from pGL3-Basic (Promega, Inc) and the *Drosophila melanogaster* tubulin promoter from the National Institute for Malaria Research, London. Restriction enzyme sites for the incorporation of oligos were added to either the 5′ (pKJ1) or 3′ (pKJ2) end of the firefly luciferase gene using QuikChange Lightning Site-Directed Mutagenesis (Agilent, Inc). Expression cassette II functions as our internal control and includes a renilla luciferase gene, SV40 late polyadenylation signal and actin promoter. The actin promoter was obtained from pAc5.1/V5-His (Invitrogen, Inc.). Digestions and ligations were carried out with New England Biolabs, Inc. (NEB) products and protocols. JM109 *Escherichia coli* cells (Promega, Inc.) were employed in transformations.

**Fig. 2:**
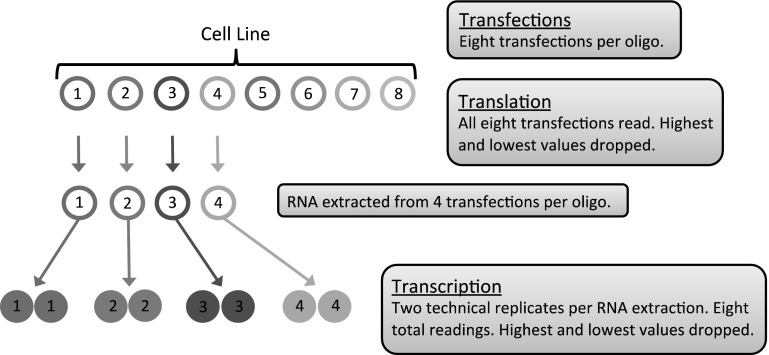
Schematic of experimental work flow

**Fig. 3:**
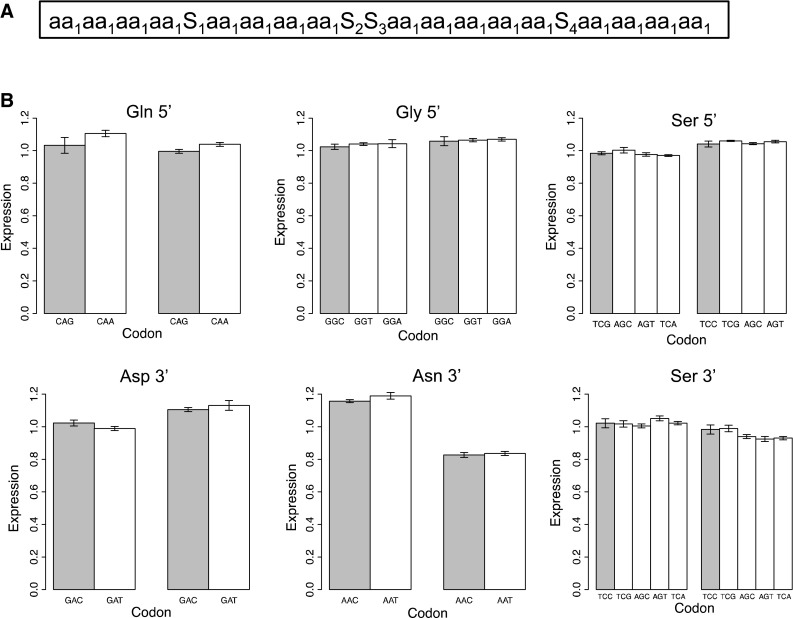
**a** Oligo used in experiments involving single amino acids. The lower case “aa_1_” represents a single amino acid with the same codon, although different experiments had different synonymous codons. *S* is an arbitrary spacer amino acid. Sequence details in Fig. S3. **b** Results of qRT-PCR for mRNA expression. The codon used (“aa_1_” above) is under the *bars*. The optimal codon (Vicario et al. 2007) is shaded. The sets of* bars* on the *left* are for the Kc167 cell line and bars on *right* the S2 cell line. There are no statistically significant differences among codons except for Ser 3′ in the S2 cell line where TCC and TCG (*left two*) differ from the AGC, AGT, and TCA (*right three*) at *p* = 0.04

Figure 2 shows the overall structure of the experiments. For testing single amino acids, we used the experimental oligo illustrated in Fig. 3a. Sixteen codons in sets of four for a single amino acid were tested with an arbitrary amino acid separating the sets of four. In all cases, direct sequencing confirmed the desired inserted sequence was in the correct position (Fig. 1b). In two cases, GGG for Gly and TCT for Ser, we were unable to insert the synthetic oligo possibly due to unfavorable secondary structures.

*Drosophila* cell lines Kc167 and S2 (obtained from the *Drosophila* Genomics Resource Center, Indiana University, Bloomington, Indiana, USA. http://dgrc.cgb.indiana.edu) were cultured in Schneider’s +10 % FBS medium. Cells were transfected with 100 ng plasmid diluted in enhancer buffer. Transfections were incubated at 25 °C for 42 h.

### Transcription

RNA was isolated using the QuickExtract RNA Extraction Kit (Epicentre, Inc.) and treated with DNase I. The SensiMix Probe One-Step Kit (BioLine, Inc.) along with single tube Custom TaqMan Gene Expression Assays (Applied Biosystems, Inc.) was utilized to carry out all real time polymerase chain reactions (RT-PCR). Reactions were run in duplicate on an Applied Biosystems 7500 Fast machine, according to the following cycling conditions: one cycle at 48 °C for 30 min, followed by 40 at 95 °C for 1 s, and 65 °C for 20 s. Means of the firefly: renilla RNA expression ratio was handled as in box in Fig. 1c.

### Translation

Luciferase protein expression was evaluated with the Dual-Glo Luciferase Assay System (Promega, Inc.) on a Modulus Microplate Luminometer (Turner BioSystems, Inc.). Twenty microlitres of Dual-Glo Luciferase Assay Reagent was added to 20 μL of each transfection and allowed to incubate at room temperature for 20 min. Levels of firefly luciferase were then measured. Twenty microlitres of Dual-Glo Stop and Glo reagent was next added to quench firefly but activate renilla luminescence. Reactions incubated at room temperature for another 20 min and levels of renilla luciferase were subsequently measured. Data were handled as in Fig. 1c.

## Results

Figure 1a illustrates the plasmid we have developed. It has two reporter genes to measure level of protein production; we used different promoters (tubulin and actin) to avoid competition for transcription factors. In one reporter gene (firefly luciferase) we incorporated cloning sites at either the 5′ or 3′ end into which we inserted an experimental oligonucleotide (Fig. 1a). *Drosophila* tissue culture cells were transfected and level of transcription of the two reporter genes were measured by quantitative reverse PCR (qRT-PCR) and level of translation measured by reading fluorescence at two different wavelengths for the firefly and renilla luciferases. In all experiments a control was run with no inserted oligo and the data handled as indicated in the box in Fig. 1c. By basing the results on the ratio of expression of the two reporters, we control for the number of plasmids taken up in any given experiment because both reporter genes must be in the same copy number. This allows us to make comparisons between transfections. Figure 2 shows the overall structure of the experiments.

We carried out experiments following the temporal dynamics of reporter gene expression and effects of plasmid quantity (Supplementary Material Figs. S1, S2) to determine an appropriate amount of plasmid to use and time of incubation post-transfection. The data presented here used 100 ng of plasmid analyzed 42 h post-transfection. Because long-term tissue culturing can induce genetic anomalies in cells, we used two *D. melanogaster* cell lines, Kc167 and S2 (*Drosophila* Genomics Resource Center, http://dgrc.cgb.indiana.edu), to mitigate against artifacts that may occur in any single cell line.

Most of the experiments reported here were performed using experimental oligos testing the effects of codons for a single amino acid and illustrated in Fig. 3a (exact sequences in Fig. S3). Sixteen codons coding for one amino acid with the same codon were in runs of four separated by arbitrary one or two codons. Optimal and non-optimal codons were defined as in Vicario et al. (2007).

Figure 3 shows results for levels of transcription as determined by qRT-PCR. As far as detectable, codon usage had little or no effect on transcription.

Levels of protein production (translation) were affected by two factors: the codon used and the strength of the secondary structure of the 5′ ribosome-binding site of mRNA, as previously shown for bacteria (Kudla et al. 2009). The minimum free energies (MFEs) of the predicted secondary structure of the 41 bp 5′ ribosome-binding site were determined by software (RNA fold, http://rna.tbi.univie.ac.at/cgi-bin/RNAFold.cgi).

**Fig. 4:**
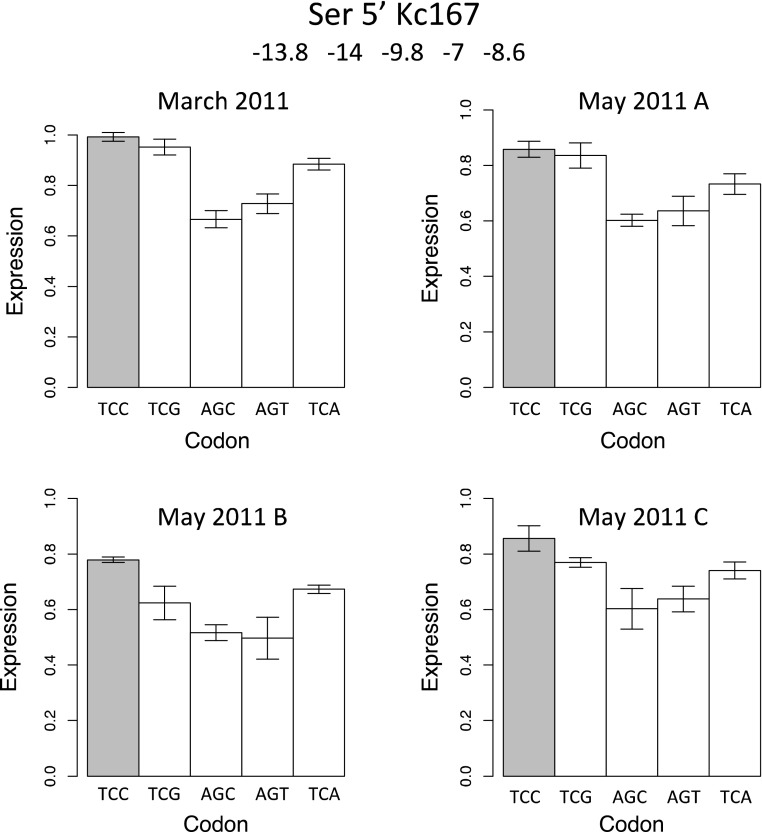
Replicate experiments for Ser codons. Shaded bar is the optimal codon. The cell line is Kc167 and the insertions are in the 5′ end. Mean free energies (kcal/mol) for the predicted secondary structure of the 41 bp 5′ ribosome binding sequences are listed in order of codons in graphs. All four replicates are independent in that they were independent transfections performed with cells taken from separate cultures and in one case three months apart. Standard errors are indicated on tops of bars

To determine robustness and repeatability of the system, we performed experiments using Ser codons in four separate experiments over several months with cells derived from independent cultures. Figure 4 shows the results. Interestingly, the two optimal codons, TCC and TCG, provide the highest level of protein expression despite having stronger secondary structure of the ribosome-binding site compared to other codons. The patterns are similar in the two cell lines. One anomaly appears to be the codon TCA which is rarely used in *D. melanogaster* yet is translated nearly as efficiently as the two optimal codons. The most abundant tRNA^Ser^ has AGA in the anticodon (Genomic tRNA database, http://gtrnadb.ucsc.edu/Dmela/). The first A (corresponding to the wobble position) undergoes a base modification to inosine; it is known that inosine translates C and A about equally efficiently (Yokoyama and Nishimura 1995). Thus if this modification is greater in the tissue culture cells than in intact flies, this would explain why TCC and TCA are translated about equally efficiently.

**Fig. 5:**
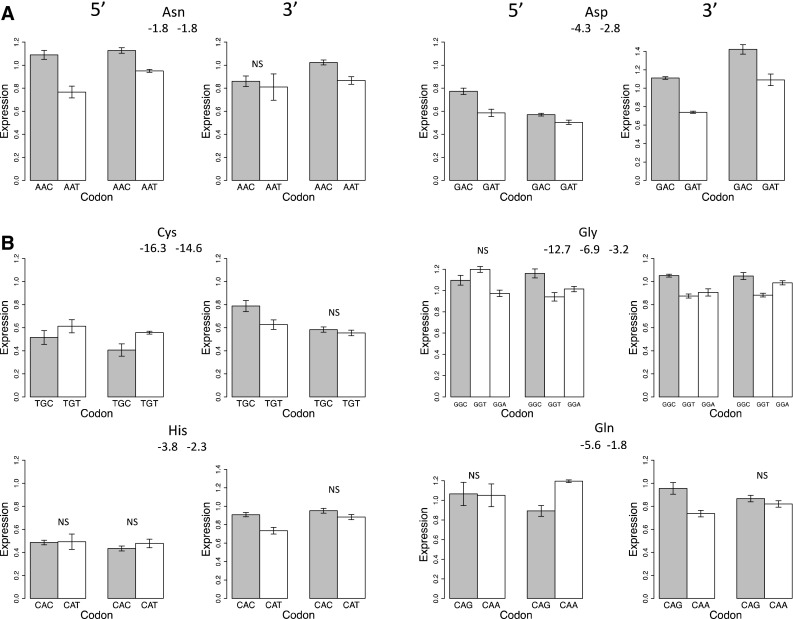
**a** Two amino acids (Asn and Asp) where expression of the optimal codon (shaded bar) is greater in both the 5′ and 3′ ends. The minimal free energies (MFEs, kcal/mol) of the 41 bp 5′ ribosome binding site is shown just below the amino acid abbreviation. **b** Four amino acids where the optimal codon (shaded bar) had a stronger secondary structure of the ribosome binding site (lower MFE) than the alternative codons and where the non-optimal codon produced higher protein expression in the 5′ end while the optimal codon produced higher protein expression in the 3′ end. In all cases, the Kc167 cell line is on left and S2 cell line on the right and the oligo is designed as in Fig. 3a. In all cases differences between amino acids is significant at *p* < 0.01 except for those with the NS indication

For two amino acids, Asn and Asp, the ribosome binding sequence for the oligo with the optimal codon in the 5′ end had stronger or equal secondary structure than the non-optimal codon, yet in both the 5′ and 3′ in both cell lines, the mRNA with optimal codons had higher translation (Fig. 5a). For four other amino acids, mRNAs with the oligo in the 5′ end with optimal codons had stronger secondary structure in the ribosome binding site than the mRNA with non-optimal codons (Fig. 5b). When in the 5′ end, mRNAs with the non-optimal codons with weaker secondary structure had higher or equal translation compared to the mRNAs with optimal codons. When moved to the 3′ end so that ribosome binding is no longer a factor, the mRNAs with optimal codons produced higher levels of protein than mRNAs with non-optimal codons.

In Figs. 4 and 5 it should be noted that in almost all cases, adding the polypeptide coded by the experimental oligo to either the 5′ or 3′ of the firefly luciferase result in a decrease in luminescence given off by the reporter, i.e., the level of luminescence is less than the control of no oligo (box in Fig. 1c). For two other amino acids we tested, Tyr and Leu, the effect of the added polypeptide on luciferase activity was too great to make reliable measurements (Supplementary Material Fig. S4).

**Fig. 6:**
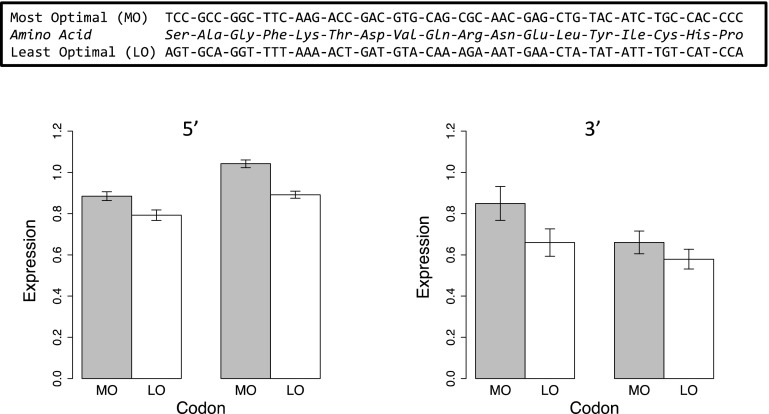
Experiment with one each of the 18 amino acids coded by more than one codon. The order was randomly determined and the oligos shown in the* box*. In this case, the MO and LO mRNA had identical MFEs of their ribosome binding sites −6.6 kcal/mol). Kc167 cell line on *left*, S2 on *right*. Differences are significant at *p* < 0.001 for 5′ experiments and *p* < 0.01 for 3′ experiments

We also tested the effect of inserting an oligo that coded for all 18 redundant amino acids; one oligo had all the optimal codons and the other all least optimal (LO) (Fig. 6). In this case the predicted MFE of the ribosome binding sites are equal (−6.6 kcal/mol). The “most optimal” (MO) was translated at higher level in both cell lines and in both the 5′ and 3′ ends compared to LO.

Statistically, we show standard errors in the figures. We also performed *t* tests and ANOVA. Statistical significance is indicated in the figures and in captions to figures.

## Discussion

These results are direct evidence consistent with a plethora of indirect evidence indicating optimal codons are translated more efficiently than non-optimal codons leading to higher protein product. These results are also consistent with what has been found in bacteria (Plotkin and Kudla 2011), namely that when codon usage in the 5′ end of messages results in differences in the strength of the secondary structure of the ribosome binding site, weaker secondary structure results in greater translation. To our knowledge, this is the first time this has been shown for a multicellular eukaryote.

The balance of the effect of secondary structure and codon usage varies among amino acids. In some cases, Ser, Asn, and Asp, even if the optimal codon in the ribosome binding site produces stronger secondary structure, protein production is greater than for the non-optimal codons (Figs. 4, 5a). In other cases (Fig. 5b) the effect of secondary structure is stronger than the effect of efficiency of translation of codons, so the effect of codon usage is dependent on position along the mRNA.

It is admittedly artificial to have four set of four identical amino acids separated by one or two codons. We designed the experiments this way to maximize the effects of codon differences for a single amino acid as we did not know how sensitive our procedure would be. It is important to emphasize that when a more realistic set of amino acids such as the oligo with one each of the redundant amino acids is tested, the results are measurable and consistent with the results when single amino acids are tested, i.e., optimal codons result in higher levels of protein expression (Fig. 6).

Quantitatively, our results compare favorably with the most relevant previous study, that of Carlini and Stephan (2003). They changed the codon for Leu from the optimal (codons naturally in the fly) to rarely used, presumed non-optimal, codons. When six or ten optimal codons in the alcohol dehydrogenase (*Adh*) gene in *Drosophila* were substituted with non-optimal codons, this resulted in a 19 and 24 % reduction in protein expression, respectively. Six and ten codons represent 2.3 and 3.9 % of the *DrosophilaAdh* gene. In our work, 16 codons represent 2.8 % of the luciferase gene. Our observed differences in expression are in the range of 5–25 %, depending on amino acid. Unfortunately, the amino acid studied by Carlini and Stephan (2003), Leu, so diminished luciferase activity as to be uninformative (Supplementary Material Fig. S4) so we could not make a more direct comparison with that previous study.

The plasmid we developed (Fig. 1a, b) is flexible in that any oligo can be inserted and the position of the experimental oligo in the expressed protein can be at either end. Having two reporters controls for efficiency of transfection, i.e., the two reporters are in the same copy number regardless of the number of plasmids taken up. Thus by treating the data as in the box in Fig. 1c, comparisons across transfections can legitimately be made. We have also tested this plasmid in a human cell line and obtained reporter expression similar to that found in *Drosophila* cells (Table S1), so potentially this plasmid can be used for similar studies in a broader range of organisms.

## Electronic supplementary material

Supplementary material 1 (DOCX 460 kb)
