# Supplementary material for: Effects of Codon Usage on Gene Expression: Empirical Studies on Drosophila
Source: J Mol Evol. 2015 Apr 3;80(3-4):219–26. doi: 10.1007/s00239-015-9675-y (PMC4408374; doi:10.1007/s00239-015-9675-y)
Supplement: Supplementary file 1 — Supplementary material 1 (DOCX 460 kb) [file 239_2015_9675_MOESM1_ESM.docx]

**Supplementary Material:**

**Figure S1.** Temporal dynamics of expression of luciferase in the transfected Drosophila cells. Experiments were performed stopping the reaction at 42 hours post-transfection.

**Figure S2**. Dilution series of amounts of plasmid used in transfections stopped at different times post-transfection.

**Figure S3.** Nucleotide sequences of inserted oligonucleotides studied.

**Figure S4.** Examples of amino acids that so decreased luciferase activity as to be non-usable.

**Table S1** Results of transfection of human cell line hESC(T) with pKJ-1 (Figures 1 and S1) with oligos illustrated in Figure 2A. Six replicate readings of firefly luciferase are shown in columns 1 to 6. Control is no oligo inserted. Serine insertions resulted in reliable readings with reduced activity compared to control comparable to that seen in Drosophila cells (Figure 3). Leucine insertions resulted in almost no activity as found for Drosophila (Figure S3).

| Codon | 1 | 2 | 3 | 4 | 5 | 6 | Mean |
| --- | --- | --- | --- | --- | --- | --- | --- |
| Control | 739 | 522 | 767 | 744 | 666 | 644 | 680 |
| **Ser** |  |  |  |  |  |  |  |
| TCG | 587 | 601 | 509 | 567 | 637 | 538 | 573 |
| TCC | 504 | 567 | 484 | 477 | 444 | 490 | 495 |
| AGT | 446 | 435 | 498 | 399 | 451 | 443 | 445 |
| TCA | 399 | 356 | 379 | 400 | 401 | 380 | 386 |
| AGC | 560 | 510 | 602 | 580 | 576 | 601 | 572 |
| **Leu** |  |  |  |  |  |  |  |
| CTA | 0.99 | 0.70 | 0.51 | 0.48 | 0.47 | 0.64 |  |
| CTG | 0.65 | 0.78 | 1.49 | 0.47 | 0.58 | 0.42 |  |
